# Earache, Chloroform Vapor

**Published:** 1880-01

**Authors:** 


### EARACHE, CHLOROFORM VAPOR.

Dr. Morgan states that he had often promptly relieved the distressing earache of children, by filling the bowl of a common new clay pipe with cotton wool, upon which he dropped a few drops of chloroform, and inserting the stem carefully into the external canal, and adjusting his lips over the bowl, blew through the pipe forcing the chloroform vapor upon the membrana tympani.—*National Medical Review.*

